# Interrelationship of *Streptococcus pneumoniae*, *Haemophilus influenzae* and *Staphylococcus aureus* colonization within and between pneumococcal-vaccine naïve mother-child dyads

**DOI:** 10.1186/1471-2334-13-483

**Published:** 2013-10-17

**Authors:** Tinevimbo Shiri, Marta C Nunes, Peter V Adrian, Nadia Van Niekerk, Keith P Klugman, Shabir A Madhi

**Affiliations:** 1 Department of Science and Technology/National Research Foundation, Vaccine Preventable Diseases, University of the Witwatersrand, Johannesburg, South Africa; 2 Medical Research Council: Respiratory and Meningeal Pathogens Research Unit, Faculty of Health Sciences, University of the Witwatersrand, Johannesburg, South Africa; 3 National Institute for Communicable Diseases: a division of National Health Laboratory Service, Centre for Respiratory and Meningitis Pathogens, 1 Modderfontein Road, Sandringham, Johannesburg, South Africa; 4 Hubert Department of Global Health, Rollins School of Public Health, and Division of Infectious Diseases, School of Medicine, Emory University, Atlanta, GA, USA

**Keywords:** Bacterial interaction, Colonization, Pneumococcal conjugate vaccine, Pneumococcus, *Staphylococcus aureus*, *Haemophilus influenzae*

## Abstract

**Background:**

A high prevalence of bacterial nasopharyngeal co-infections has been reported in children, however, such data is limited in adults. We examined the interaction of *Haemophilus influenzae*, *Staphylococcus aureus* and *Streptococcus pneumoniae* pharyngeal colonization in mother-child dyads.

**Methods:**

Pneumococcal-vaccine naïve children and their mothers had pharyngeal swabs undertaken at 1.6, 2.5, 3.5, 4.5, 7.4, 9.5, 12.5, 16.2 and 24.2 months of child’s age. Swabs were cultured for *S*. *pneumoniae*, *H*. *influenzae* and *S*. *aureus* using standard microbiologic methods. Multivariate generalized estimating equation-models were used to explore the associations of the three bacteria within and between children and their mothers.

**Results:**

In children, the observed probability of co-colonization was higher than expected. Well-defined associations in colonization between the bacteria were observed in children but not among mothers. In children, a synergistic association was observed between *S*. *pneumoniae* and *H*. *influenzae* (Adjusted odds ratio (AOR): 1.75, 95% CI: 1.32-2.32) and a negative association between *S*. *pneumoniae* and *S*. *aureus* (AOR: 0.51, 95% CI: 0.39-0.67) or *H*. *influenzae* and *S*. *aureus* (AOR: 0.24, 95% CI: 0.16-0.34) colonization. Additionally, all three bacteria had a higher likelihood of concurrent colonization. There was a strong association in colonization by the bacteria in children and their mothers, including increased likelihood of maternal colonization if the child was colonized by *S*. *pneumoniae* (AOR: 1.84, 95% CI: 1.28-2.63) and *H*. *influenzae* (AOR: 6.34, 95% CI: 2.24-18.0).

**Conclusions:**

The effects of immunization of children with pneumococcal-conjugate-vaccine in settings such as ours needs monitoring with regard to potential changes of pharyngeal bacterial ecology which could occur in vaccinated and –unvaccinated age-groups.

## Background

*Streptococcus pneumoniae* is commonly associated with colonization of the nasopharynx (NP) in children, with variable co-colonization by other bacteria such as *Staphylococcus aureus*, *Moraxella catarrhalis* and *Haemophilus influenzae*[1-5]. Although generally asymptomatic, colonization by these bacteria is part of the pathogenesis to developing invasive and mucosal disease [6].

A negative association for colonization between *S*. *pneumoniae* and *S*. *aureus*; and *H*. *influenzae* and *S*. *aureus* has been reported in otherwise healthy children [1,7-9], but not so among asymptomatic and hospitalized human immunodeficiency virus (HIV)-infected children [10,11]. Furthermore, a positive association has been observed for *H*. *influenzae* and *S*. *pneumoniae* colonization among children, irrespective of HIV-infection status [10-12].

There are limited studies on nasopharyngeal bacterial colonization associations within and between mother and infant pairs. Lebon et al. reported an association for colonization with *S*. *aureus* and *H*. *influenzae*, but not *S*. *pneumoniae* and *M*. *catarrhalis*, between mothers and their children [13]. Bacterial interactions in the NP can be altered by treatments and vaccines [14]. Previous studies have demonstrated subsequent changes in other bacteria following the introduction of pneumococcal conjugate vaccine (PCV) [15-18]. Understanding the nasopharyngeal interaction of these bacteria within and between mothers and their children could assist in predicting the potential impact of PCV immunization on the ecology of nasopharyngeal bacterial colonization and transmission patterns of these bacteria.

The aim of this study was to determine the association of maternal colonization with *S*. *pneumoniae*, *H*. *influenzae* and *S*. *aureus* and colonization with the same bacteria in children. In addition, we explored the correlation of colonization statuses of mothers and children.

## Methods

### Ethics statement

This study was approved by the Human Research Ethics Committee (HREC number: 050705) at the University of the Witwatersrand, Johannesburg, South Africa. Signed informed consent for collection of the isolates was obtained from the mothers.

### Study design

The data sets were derived from a cohort study in Soweto, South Africa, involving infants and their mothers as described in Nunes et al. 2013 [19]. Briefly, 251 PCV-naïve infants 6–12 weeks of age and their mothers were followed until two years of age between January 2007 and May 2009. Infants were stratified as being: i) born to HIV-infected mothers, but who were HIV-uninfected themselves based on a negative HIV-polymerase chain reaction (Roche Amplicor RNA PCR, version 1.5, Roche Molecular Systems, Inc. Branchburg, NJ, USA) at baseline and in the absence of being breast-fed (HEU); and ii) infants born to mothers who were sero-negative for HIV after 24 weeks of gestational age during pregnancy and the infant was also sero-negative for HIV at enrolment (HUU).

Nasopharyngeal sampling for culture of *Streptococcus pneumoniae*, *Staphylococcus aureus* and *Haemophilus influenzae* carriage were undertaken in the mother-child dyad at 1.6, 2.5, 3.5, 4.5, 7.4, 9.5, 12.5, 16.2, and 24.2 months of the child’s chronological age. In addition, oropharyngeal swabs were also undertaken for bacterial culture among the mothers. Infants received all their scheduled childhood vaccines which included *Haemophilus influenzae* type b conjugate vaccine, while PCV was unavailable at the time of the study. Swabs were cultured for pneumococci according to standard methods [20] and serotyped by the Quellung method (Statens Serum Institute, Copenhagen, Denmark) at the National Institute for Communicable Diseases, South Africa. Serotype 6C was distinguished from serotype 6A by PCR [21]. Strains which did not react by the Quellung method were confirmed as being pneumococci by *lyt*A PCR detection and categorized as non-typeable. Pneumococcal isolates were categorized as PCV13 serotypes (1, 3, 4, 5, 6A, 6B, 7F, 9V, 14, 18C, 19A, 19F, 23F) or non-PCV13 serotypes (all other serotypes and non-typeable isolates). Colonization for *S*. *pneumoniae*, *S*. *aureus* and *H*. *influenzae* as a binary variable was established for each time point. Data from all the children and the mothers with at least one sample were included in the analysis.

### Statistical analyses

A Mann-Kendall test for detecting trends was used to examine the relationship between participant age and the prevalence of bacteria. Patterns of possible associations of the three bacteria were analyzed by fitting generalized estimating equations (GEE) models with an exchangeable correlation structure and the logit link function, i.e. we modelled colonization by each bacterium separately with colonization of the other two bacterial species, including the interaction of these two other bacteria, as independent variables. Also, age of the study participant, influenza virus season, season of the year when the sample was taken, presence of a child in the household between the ages of 3 and 6 years, use of coal/wood for fuel in the household, presence of a sibling attending day care and presence of a smoker in the household were included in the model to adjust for potential confounding. Participants who were not colonized by pneumococcus were used as the reference group in our GEE models. Adjusted odds ratios (AOR) together with 95% confidence intervals (95% CI) were used as measures of association. All statistical analyses were performed with SAS version 9.2 software (SAS Institute, Inc., NC, USA).

## Results

A total of 1844 samples from 251 children (median 8 samples per child) were obtained, including 983 from 126 HEU children and 861 samples from 125 HUU children. Similarly, 1835 samples from 251 mothers (median 8 per mother) were evaluated, including 985 samples from 126 HIV-infected mothers and 850 samples from 125 HIV-uninfected mothers. A total of 1830 specimens from 251 mother-child pairs had concurrent samples at the same visit.

**Figure 1:**
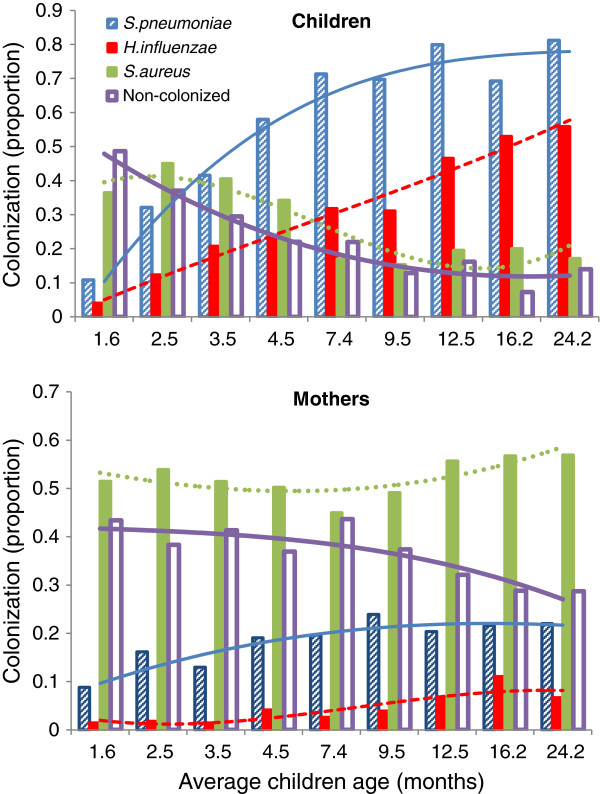
**Prevalence of *S***. ***pneumoniae***, ***H***. ***influenzae *and *S***. ***aureus *in children and mothers.** Proportion of samples positive for *S*. *pneumoniae*, *H*. *influenzae* and *S*. *aureus* in children (top) and mothers (bottom) stratified by child age, represented by bars. Cubic functions, represented by lines, giving prevalence trends are shown for each bacterium.

The prevalence of bacterial colonization at individual time points in both children and mothers are shown in Figure 1 (see also Additional file 1: Table S1). In children, there was an upward trend in prevalence of *H*. *influenzae* (p = 0.0003) and *S*. *pneumoniae* (p = 0.002) colonization with increasing age, while there was a decline in *S*. *aureus* colonization (p = 0.02) with age. Among mothers, the prevalence of *S*. *pneumoniae* (p = 0.002) and *H*. *influenzae* (p = 0.008) colonization also increased over time as the child became older, however, no changes were observed in prevalence of *S*. *aureus* colonization which remained greater than 50% throughout the study (Figure 1).

### Bacterial colonization associations in children

**Figure 2:**
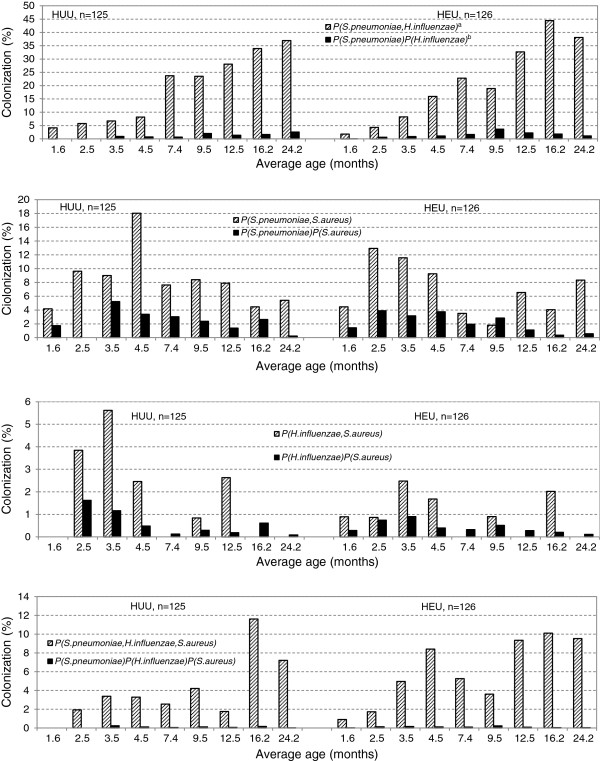
**Comparison of observed and calculated prevalence in children.** Observed prevalence of dual and triple carriage in children (i.e. pattern filled bars) and calculated prevalence given by random models (i.e. solid filled bars). Here HEU means HIV-uninfected children born to HIV-infected mothers and HUU mean HIV-uninfected children born to HIV-uninfected mothers. ^a^P (*S.pneumoniae, H.influenzae*) - observed probability that a child carried both *S. pneumoniae* and *H. influenzae*. ^b^P (*S. pneumoniae*) P (*H. influenzae*) – calculated (or expected) probability of dual carriage under the assumption that co-carriage is an independent process

The overall detection of colonization in children was 59.8% (1094/1830) for *S*. *pneumoniae*, 32.3% (591/1830) for *H*. *influenzae* and 26.6% (487/1830) for *S*. *aureus*. The observed probability of bacterial co-colonization, either dual or triple, at a particular time-point was higher than expected (calculated from the assumption that colonization was an independent process and not random), i.e. pathogens were found together more often than would be expected by chance as shown in Figure 2. Carriage of *H*. *influenzae* increased the odds of subsequent colonization with *S*. *pneumoniae* (AOR: 1.75, 95% CI: 1.32-2.32), but not vice-versa (AOR: 1.14, 95% CI: 0.90-1.44) (Table 1). There was a greater likelihood of childhood colonization by *H*. *influenzae* if colonized by PCV13 serotypes (AOR: 1.45, 95% CI: 1.09-1.93) and vice-versa, i.e., increased likelihood of colonization by PCV13 serotypes if colonized by *H*. *influenzae* (AOR: 1.75, 95% CI: 1.28-2.39). A similar, albeit non-significant trend was also observed for colonization between *H*. *influenzae* and non-PCV13-serotypes (AOR: 1.35, 95% CI: 0.96-1.90) and vice-versa (AOR: 1.36, 95% CI: 0.96-1.91).

**Table 1:** **Interaction between *Streptococcus pneumoniae***, ***Haemophilus influenzae *and *Staphylococcus aureus *nasopharyngeal colonization in children** <**2 years of age**

**Independent variable**	**Dependent variable**				
	***S***. ***pneumoniae***			***H***. ***influenzae***	***S***. ***aureus***
	*S*. *pneumoniae*	PCV13^b^	Non-PCV13^c^		
*S*. *pneumoniae* colonization^a^	-	-	-	1.14(0.90,1.44)	0.68(0.50,0.93)
PCV13	-	-	-	1.45(1.09,1.93)	0.74(0.52,1.06)
Non-PCV13	-	-	-	1.35(0.96,1.90)	0.62(0.42,0.92)
*H*. *influenzae* colonization	1.75(1.32,2.32)	1.75(1.28,2.39)	1.36(0.96,1.91)	-	0.58(0.34,0.98)
*S*. *aureus* colonization	0.51(0.39,0.67)	0.48(0.36,0.65)	0.39(0.29,0.54)	0.24(0.16,0.34)	-
Age of child	1.09(1.06,1.10)	1.08(1.05,1.10)	1.05(1.02,1.07)	1.05(1.03,1.07)	0.95(0.93,0.96)
Influenza season (1-influenza season, 0-peri influenza season)	1.37(1.06,1.77)	1.46(1.11,1.93)	1.45(1.05,1.98)	1.54(1.21,1.96)	0.93(0.70,1.24)
Season of the year	0.88(0.80,0.97)	0.80(0.72,0.89)	0.74(0.66,0.82)	0.69(0.63,0.76)	0.86(0.78,0.95)
Any child between 3–6 years in the household (y/n)	1.19(0.92,1.56)	1.06(0.78,1.44)	0.99(0.71,1.38)	0.89(0.68,1.16)	0.94(0.68,1.29)
Use of Coal/Wood for fuel (y/n)	1.58(0.98,2.54)	1.14(0.64,2.03)	1.43(0.73,2.82)	0.49(0.26,0.90)	1.49(0.84,2.65)
Child attending day care (y/n)	0.83(0.59,1.16)	0.84(0.59,1.19)	0.84(0.55,1.28)	1.51(1.09,2.08)	1.34(0.99,1.83)
Any smoker in the household (y/n)	0.94(0.70,1.26)	0.79(0.57,1.08)	0.85(0.61,1.19)	0.76(0.58,1.02)	1.13(0.86,1.49)
**Bacterial interactions**					
*H*. *influenzae* and *S*. *aureus*	2.28(1.31,3.97)	1.90(1.05,3.45)	4.16(2.24,7.73)	-	-
*H*. *influenzae* and *S*. *pneumoniae*	-	-	-	-	1.74(0.95,3.17)
*S*. *pneumoniae* and *S*. *aureus*	-	-	-	3.59(2.24,5.76)	-
*H*. *influenzae* and PCV13-serotype	-	-	-	-	1.37(0.70,2.67)
*S*. *aureus* and PCV13-serotype	-	-	-	2.61(1.51,4.51)	-
*H*. *influenzae* and non-PCV13-serotype	-	-	-	-	2.50(1.27,4.91)
*S*. *aureus* and non-PCV13-serotype	-	-	-	4.87(2.60,9.14)	-

The table shows the odds of carriage (and 95% confidence intervals; 95%CI) of a particular bacterium given the presence/absence of other pathogens adjusted for other risk factors. Subjects who were not colonized by any pneumococci were the reference group. Seasons of the year were categorized as Summer (December–February), Autumn (March–May), Winter (June–August) and Spring (September–November). The timing of the influenza epidemic was determined according to the national influenza surveillance program conducted at the National Institute for Communicable Diseases.

^a^All pneumococci, ^b^PCV13 – serotypes included in the thirteen-valent pneumococcal conjugate vaccine, i.e. serotypes 1, 3, 4, 5, 6A, 6B, 7F, 9V, 14, 18C, 19A, 19F and 23F. ^c^Non-PCV13- all serotypes not included in PCV13.

Colonization by *S*. *pneumoniae* was associated with a reduced odds of concurrent *S*. *aureus* colonization (AOR: 0.68, 95% CI: 0.50-0.93) and vice-versa (AOR: 0.51, 95% CI: 0.39-0.67). This was not differentially affected by pneumococcal PCV13-serotype status. When stratified according to PCV13-serotypes, carriage of *S*. *aureus* decreased the odds of colonization by PCV13-serotypes (AOR: 0.48, 95% CI: 0.36-0.65) and non-PCV13-serotype (AOR: 0.39, 95% CI: 0.29-0.54) (Table 1).

There was a negative association between colonization by *H*. *influenzae* and *S*. *aureus*; AOR: 0.24, 95% CI: 0.16-0.34 and vice-versa (AOR: 0.58, 95% CI: 0.34-0.98). Furthermore, co-carriage of either PCV13-serotypes and *S*. *aureus* or non-PCV13-serotypes and *S*. *aureus* increased the odds of colonization by *H*. *influenzae* (AOR: 2.61, 95% CI: 1.51-4.51 and AOR: 4.87, 95% CI: 2.60-9.14, respectively). Concurrent carriage of *H*. *influenzae* and *S*. *aureus* increased the odds of colonization by *S*. *pneumoniae* (AOR: 2.28, 95% CI: 1.31-3.97), including colonization by PCV13-serotypes (AOR: 1.90, 95% CI: 1.05-3.45) and non-PCV13-serotypes (AOR: 4.16, 95% CI: 2.24-7.33). Thus, competitive interactions between *H*. *influenzae* and *S*. *aureus* or *S*. *pneumoniae* and *S*. *aureus* shifted to synergistic when concurrent *S*. *pneumoniae* and *H*. *influenzae* carriage were present. There was no association between carriage of *S*. *aureus* given dual carriage of *S*. *pneumoniae* and *H*. *influenzae* (AOR: 1.71, 95% CI: 0.95-3.06) (Table 1). Of the other risk factors, age and influenza virus season were positively associated with colonization by both *S*. *pneumoniae* and *H*. *influenzae*. The presence of a child attending day care in the household increased the odds of colonization by *H*. *influenzae* (AOR: 1.51, 95% CI: 1.09-2.08).

### Bacterial interactions in mothers

Overall detection of colonization in mothers across all study visits were 18.9% (345/1830) for *S*. *pneumoniae*, 4.5% (83/1830) for *H*. *influenzae* and 52.1% (953/1830) for *S*. *aureus*. No interactions were observed between the three bacteria in mothers, even when analyzing the composite data of HIV-infected and -uninfected mothers (Table 2). This was also true even if the women were stratified by HIV-infection status (data not shown). Age of the mother was negatively associated with colonization by *S*. *pneumoniae* (AOR: 0.94, 95% CI: 0.93-0.95) and *H*. *influenzae* (AOR: 0.93, 95% CI: 0.91-0.96), and positively associated with colonization by *S*. *aureus* (AOR: 1.02, 95% CI: 1.01-1.03).

**Table 2:** **Interaction between *Streptococcus pneumoniae***, ***Haemophilus influenzae *and *Staphylococcus aureus *nasopharyngeal colonization in mothers**

**Independent variable**	**Dependent variable**				
	***S***. ***pneumoniae***			***H***. ***influenzae***	***S***. ***aureus***
	*S*. *pneumoniae*	PCV13^b^	Non-PCV13^c^		
*S*. *pneumoniae* colonization^a^	-	-	-	1.14(0.56,2.30)	0.83(0.63,1.10)
PCV13	-	-	-	0.79(0.23,2.68)	0.85(0.60,1.21)
Non-PCV13	-	-	-	1.49(0.65,3.45)	0.81(0.57,1.16)
*H*. *influenzae* colonization	1.48(0.74,2.97)	0.97(0.31,3.07)	2.03(0.91,4.54)	-	1.04(0.64,1.69)
*S*. *aureus* colonization	0.77(0.57,1.03)	0.76(0.52,1.12)	0.72(0.51,1.03)	0.66(0.40,1.08)	-
Age of the mother	0.94(0.93,0.95)	0.91(0.90,0.93)	0.93(0.91,0.95)	0.93(0.91,0.96)	1.02(1.01,1.03)
Influenza season (1-influenza season, 0-peri influenza season)	1.00(0.75,1.33)	0.96(0.65,1.42)	1.06(0.72,1.55)	1.52(0.85,2.74)	0.87(0.70,1.09)
Season of the year	1.16(1.01,1.32)	1.19(0.99,1.43)	1.05(0.89,1.24)	0.64(0.47,0.88)	0.88(0.80,0.98)
Any child between 3–6 years in the household (y/n)	0.72(0.51,1.02)	0.73(0.45,1.19)	0.70(0.45,1.09)	1.09(0.59,2.01)	0.80(0.59,1.08)
Use of Coal/Wood for fuel (y/n)	1.56(0.91,2.67)	1.40(0.65,3.01)	1.49(0.71,3.12)	0.66(0.09,5.04)	0.88(0.44,1.76)
Any child attending day care in the household (y/n)	1.20(0.89,1.62)	1.37(0.89,2.10)	1.01(0.68,1.51)	1.02(0.54,1.90)	1.10(0.83,1.47)
Any smoker in the household (y/n)	0.99(0.71,1.39)	1.00(0.64,1.57)	1.02(0.68,1.54)	0.81(0.48,1.35)	0.93(0.70,1.25)
**Bacterial interactions**					
*H*. *influenzae* and *S*. *aureus*	1.50(0.54,4.17)	1.85(0.38,9.09)	1.34(0.41,4.42)	-	-
*H*. *influenzae* and *S*. *pneumoniae*	-	-	-	-	1.20(0.50,2.89)
*S*. *pneumoniae* and *S*. *aureus*	-	-	-	1.97(0.71,5.46)	-
*H*. *influenzae* and PCV13-serotype	-	-	-	-	1.55(0.36,6.64)
*S*. *aureus* and PCV13-serotype	-	-	-	2.33(0.49,11.0)	-
*H*. *influenzae* and non-PCV13-serotype	-	-	-	-	1.16(0.45,2.96)
*S*. *aureus* and non-PCV13-serotype	-	-	-	1.81(0.55,6.01)	-

The table shows the odds of carriage (and 95% confidence intervals; 95%CI) of a particular bacterium given the presence/absence of other pathogens adjusted for other risk factors. Subjects who were not colonized by any pneumococci were the reference group. Seasons of the year were categorized as Summer (December–February), Autumn (March–May), Winter (June–August) and Spring (September–November). The timing of the influenza epidemic was determined according to the national influenza surveillance program conducted at the National Institute for Communicable Diseases ^a^All pneumococci, ^b^PCV13 – serotypes included in the thirteen-valent pneumococcal conjugate vaccine, i.e. serotypes 1, 3, 4,5, 6A, 6B, 7F, 9V, 14, 18C, 19A, 19F and 23F, ^c^Non-PCV13-all serotypes not included in PCV13.

### Colonization correlation statuses of mothers and children

The proportion of children who ever carried *S*. *pneumoniae* during the study period was one and half-fold greater compared to mothers (95.6% vs. 66.1%; p < 0.0001), three-fold greater for *H*. *influenzae* (84.1% vs. 26.3%; p < 0.0001), while the proportion of children ever colonized by *S*. *aureus* was 15% lower than that of mothers (74.9% vs. 88.1%; p = 0.0001). We explored the colonization correlation statuses of mothers and children, i.e., the interrelationship of the three bacteria in mothers taking into account bacterial colonization in the children, and vice-versa with the results given in Table 3.

**Table 3:** Colonization correlation statuses of mothers and children

**Dependent variable**	**Independent variables**											
	**Carriage in children**						**Carriage in mothers**					
	*S*.*pneumoniae*	*H*.*influenzae*	*S*. *aureus*	*S*.*pneumoniae* and	*S*.*pneumoniae* and	*H*.*influenzae* and	*S*.*pneumoniae*	*H*.*influenzae*	*S*.*aureus*	*H*.*influenzae* and	*S*.*pneumoniae* and	*S*.*pneumoniae* and
				*H*.*influenzae*	*S*.*aureus*	*S*.*aureus*				*S*.*aureus*	*H*.*influenzae*	*S*.*aureus*
**Colonization in children**												
*S*. *pneumoniae*	-	2.59	0.48	-	-	1.83	1.57	1.70	1.16	0.95	1.96	1.09
		1.97–3.40	0.36–0.64			1.11–3.44	1.04–2.39	0.65–4.45	0.90–1.51	0.27–3.32	0.53–7.23	0.59–2.01
*H*. *influenzae*	2.88		0.49		1.67		1.04	2.43	0.71	3.31	0.48	1.37
	2.24–3.70		0.30–0.79		0.94–2.96		0.73–1.50	1.19–4.97	0.56–0.90	1.26–8.69	0.16–1.47	0.85–2.21
*S*. *aureus*	0.50	0.45	-	1.91	-	-	1.03	1.11	1.65	0.71	0.78	1.09
	0.38–0.66	0.27–0.75		1.06–3.43			0.70–1.54	0.44–2.82	1.28–2.13	0.25–2.05	0.22–2.71	0.64–1.87
**Colonization in mothers**												
*S*. *pneumoniae*	1.84	1.41	1.02	0.83	1.18	0.77		1.22	0.79	1.37		
	1.28–2.63	0.81–2.48	0.62–1.69	0.45–1.53	0.63–2.21	0.41–1.44		0.62–2.43	0.60–1.05	0.53–3.52		
*H*. *influenzae*	1.67	6.34	0.35	0.55	4.49	0.56	1.38	-	1.06	-	-	1.14
	0.69–4.07	2.24–18.0	0.07–1.87	0.18–1.70	0.87–23.3	0.19–1.66	0.69–2.79		0.62–1.82			0.45–2.88
*S*. *aureus*	1.19	0.80	1.56	1.12	0.98	0.87	0.80	1.07			1.22	
	0.92–1.53	0.54–1.17	1.13–2.17	0.73–1.72	0.61–1.57	0.55–1.40	0.62–1.04	0.68–1.69			0.54–2.72	

Association of colonization with all pneumococci with *H*. *influenzae* and *S*. *aureus*.

Colonization statuses of children and mothers were correlated, i.e. carriage of a particular bacterium by the mother increased the odds of carriage of the same bacterium by the child, and vice versa. Carriage of *S*. *pneumoniae*, *H*. *influenzae* and *S*. *aureus* by mothers increased the odds of colonization in children (AOR: 1.57, 95% CI: 1.04-2.39; AOR: 2.43, 95% CI: 1.19-4.97 and AOR: 1.65, 95% CI: 1.28-2.13, respectively) (Table 3). Similarly, carriage of *S*. *pneumoniae*, *H*. *influenzae* and *S*. *aureus* by children increased the odds of colonization in mothers (AOR: 1.84, 95% CI: 1.28-2.63; AOR: 6.34, 95% CI: 2.24-18.0 and AOR: 1.56, 95% CI: 1.13-2.17, respectively). Observations were similar for PCV13 and non-PCV13 serotypes (data not shown). In this combined full mixing model, the interrelationships of the three bacteria in children were preserved, i.e. synergy and antagonistic effects of bacteria were observed in children while there were no relationships in mothers when analysis was confined to individual populations, i.e. children or mothers group separately.

## Discussion

In this analysis we explored the association of *S*. *pneumoniae*, *H*. *influenzae* and *S*. *aureus* colonization within and between children and their mothers. In children, the probabilities of dual and triple colonization were identified concurrently more often than would be expected by chance. We observed well-defined relationships of the three bacteria in children. Our results showed that exposure to maternal-HIV in these children did not change the colonization patterns by the three bacteria. There is limited data on bacterial interactions in the nasopharynx of adults; and unlike in children no interactions between colonizing bacteria were observed in the mothers. Colonization with the three bacteria in children and mothers was positively associated.

Our results among children corroborate that there is a synergistic relationship between *S*. *pneumoniae* and *H*. *influenzae* colonization and a negative association between *S*. *pneumoniae* and *S*. *aureus*, or *H*. *influenzae* and *S*. *aureus* colonization [1,7-10,15]. Further evidence of interaction of bacteria in the nasopharynx are evident from animal-model studies in which *H*. *influenzae* colonization was more likely when preceded by either *S*. *aureus* or *S*. *pneumoniae* colonization, while under some conditions *H*. *influenzae* limited colonization by *S*. *pneumoniae*[22].

In children, prior co-colonization by *S*. *pneumoniae* and *S*. *aureus* increased the risk of acquiring *H*. *influenzae*, while dual carriage of *H*. *influenzae* and *S*. *aureus* showed a trend for increasing risk for *S*. *pneumoniae* colonization. Thus, the additional presence of *H*. *influenzae* or *S*. *pneumoniae* might alter the competitive balance between *S*. *aureus* and *S*. *pneumoniae* or *H*. *influenzae* and all three pathogens are able to colonize simultaneously as was also reported by Pettigrew et al. [23]. These relations were not differentially affected by pneumococcal serotype colonization status. Thus, the cause, effect and consequences of these bacterial interactions in the nasopharynx warrants further study.

The well-defined associations observed in children were, however, not detected in the mothers, even when mothers were stratified by HIV-infection status. This suggests that bacterial interactions in the nasopharynx may be affected by biological factors of the bacteria, coupled with host immunity factors which mature with age, as well as possibly other environmental factors [15]. We observed that carriage of a particular bacterium by the mother was a risk factor for the child, and vice-versa suggesting that direct transmission between the two groups may play a role in bacterial colonization among them. This result, similar to a previous study [13], shows that nasopharyngeal colonization status in mothers with any of the three bacteria was correlated with colonization status of their children.

Though we established well defined relationships of these bacteria in children and lack of thereof in adult woman, our study has some limitations. First, we only sampled NP swabs in the children, which may underestimate prevalence of *S*. *aureus* colonization in children. Also, we did not undertake genotyping of the *S. aureus* strains and the other two bacteria, therefore we were unable to address whether the *S*. *aureus* transmission was between the mothers and children or from the environment. Furthermore, our study was not designed to address disease caused by these pathogens, but rather focussed on colonization by these pathogens. In spite of these limitations, this work provide pre-PCV immunization data which may serve as a reference to follow-up studies to determine what effect childhood PCV immunization may have on bacterial ecology of the nasopharynx.

## Conclusions

The results of this study suggest that before pneumococcal conjugate vaccine introduction, bacterial co-infections are more important in young children than in adults. Further studies are required to evaluate the effect of childhood PCV immunization on the potential changes of pharyngeal bacterial ecology and disease in vaccinated and unvaccinated populations.

## Competing interests

SAM and KPK have received grants from Pfizer and GSK and have also served on advisory boards for Pfizer, GSK, Novartis and MERCK on pneumococcal vaccines. TS, MCN, PVA, NvN declare no conflict of interests.

## Authors’ contributions

SAM and KPK were involved in the conception and design of the study, acquisition of data, analysis, and interpretation of data and critically revised the manuscript. PVA and NvN participated in the design of study, acquisition and analysis of data. TS, MCN were involved in drafting the manuscript, statistical analysis and interpretation of data. All authors read and approved the final manuscript.

## Pre-publication history

The pre-publication history for this paper can be accessed here:

http://www.biomedcentral.com/1471-2334/13/483/prepub

## Supplementary Material

Additional file 1: Table S1 Numbers of children and mothers with single, dual, triple carriage or no carriage, further stratified by HIV-exposure or maternal HIV-infection status. Click here for file

## References

[B1] Kwambana BA Barer MR Bottomley C Adegbola RA Antonio M Early acquisition and high nasopharyngeal co-colonisation by Streptococcus pneumoniae and three respiratory pathogens amongst Gambian new-borns and infants BMC Infect Dis 2011 13 175 10.1186/1471-2334-11-175 21689403 PMC3129300

[B2] Faden H Duffy L Wasielewski R Wolf J Krystofik D Tung Y Relationship between nasopharyngeal colonization and the development of otitis media in children Pediatr Infect Dis J 1997 13 132 135 10.1086/516477 9180184

[B3] Labout JAM Duijts L Arends L Jaddoe VW Hofman A De Groot R Risk factors for pneumococcal carriage in healthy Dutch infants. The Generation R study J Pediatr 2007 13 771 776 10.1016/j.jpeds.2008.05.061 18621390

[B4] Neto AS Lavado P Flores P Dias R Pessanha MA Sousa E Palminha JM Canica M Esperanca-Pina J Risk factors for the nasopharyngeal carriage of respiratory pathogens by Portuguese children: phenotype and antimicrobial susceptibility of Haemophilus influenzae and Streptococcus pneumoniae Microb Drug Resist 2003 13 99 108 10.1089/107662903764736409 12705689

[B5] Vives M Garcia ME Saenz P Mora MA Mata L Sabharwal H Svanborg C Nasopharyngeal colonization in Costa Rican children during the first year of life Pediatr Infect Dis J 1997 13 852 858 10.1097/00006454-199709000-00007 9306479

[B6] Simell B Auranen K Kayhty H Goldblatt D Dagan R O'Brien KL (PneumoCarr) ftPCG The fundamental link between pneumococcal carriage and disease Exp Rev Vacc 2012 13 841 855 10.1586/erv.12.53 22913260

[B7] Bogaert D Van Belkum A Sluijter M Luijendijk A De Groot R Rumke CH Verbrugh AH Hermans PWM Colonisation by *Streptococcus pneumoniae* and *Staphylococcus aureus* in healthy children Lancet 2004 13 1871 1872 10.1016/S0140-6736(04)16357-5 15183627

[B8] Quintero B Araque M van der Gaast-de Jongh C Escalona F Correa M Morillo-Puente S Vielma S Hermans PW Epidemiology of *Streptococcus pneumoniae* and *Staphylococcus aureus* colonization in healthy Venezuelan children Eur J Clin Microbiol Infect Dis 2011 13 7 19 10.1007/s10096-010-1044-6 20803226 PMC2998637

[B9] Regev-Yochay G Dagan R Raz M Carmeli Y Shainberg B Derazne E Rahav G Rubinstein E Association between carriage of *Streptococcus pneumoniae* and *Staphylococcus aureus* in children JAMA 2004 13 716 720 10.1001/jama.292.6.716 15304469

[B10] Madhi SA Adrian P Kuwanda L Cutland C Albrich WC Klugman KP Long-term effect of pneumococcal conjugate vaccine on nasopharyngeal colonization by Streptococcus pneumoniae–and associated interactions with Staphylococcus aureus and Haemophilus influenzae colonization–in HIV-Infected and HIV-uninfected children J Infect Dis 2007 13 1662 1666 10.1086/522164 18008250

[B11] McNally ML Jeena MP Gajee K Sturm WA Tomkins MA Coovadia MH Goldblatt D Lack of association between the nasopharyngeal carriage of *Streptococcus pneumoniae* and *Staphylococcus aureus* in HIV-1 infected South African Children J Infect Dis 2006 13 385 390 10.1086/505076 16826488

[B12] McNally ML Jeena MP Gajee K Sturm WA Tomkins MA Coovadia MH Goldblatt D Lack of interference between Streptococcus pneumoniae and Staphylococcus aureus in HIV-infected individuals J Infect Dis 2006 13 1617 1618 10.1086/508886 17083049

[B13] Lebon A Moll HA Tavakol M Van Wamel WJ Jaddoe VW Hofman A Verbrugh HA Van Belkum A Correlation of bacterial colonization status between mother and child: the Generation R Study J Clin Microbiol 2010 13 960 962 10.1128/JCM.01799-09 19940045 PMC2832455

[B14] Xu Q Almudervar A Casey JR Pichichero ME Nasopharyngeal bacterial interactions in children Emerg Infect Dis 2012 13 1738 1745 10.3201/eid1811.111904 23092680 PMC3559157

[B15] Van Gils EJ Hak E Veenhoven RH Rodenburg GD Bogaert D Bruin JP Van Alphen L Sanders EA Effect of seven-valent pneumococcal conjugate vaccine on Staphylococcus aureus colonisation in a randomised controlled trial PloS One 2011 13 e20229 10.1371/journal.pone.0020229 21695210 PMC3112202

[B16] Casey JR Pichichero ME Changes in frequency and pathogens causing acute otitis media in 1995–2003 Pediatr Infect Dis J 2004 13 824 828 10.1097/01.inf.0000136871.51792.19 15361720

[B17] Wiertsema SP Kirkham LA Corscadden KJ Mowe EN Bowman JM Jacoby P Francis R Vijayasekaran S Coates HL Riley TV Richmond P Predominance of nontypeable Haemophilus influenzae in children with otitis media following introduction of a 3 + 0 pneumococcal conjugate vaccine schedule Vaccine 2011 13 5163 5170 10.1016/j.vaccine.2011.05.035 21621576

[B18] Stamboulidis K Chatzaki D Poulakou G Ioannidou S Lebessi E Katsarolis I Sypsa V Tsakanikos M Kafetzis D Tsolia MN The impact of the heptavalent pneumococcal conjugate vaccine on the epidemiology of acute otitis media complicated by otorrhea Pediatr Infect Dis J 2011 13 551 555 10.1097/INF.0b013e31821038d9 21297521

[B19] Nunes MC Shiri T Van Niekerk N Cutland CL Groome MJ Koen A Von Gottberg A De Gouveia L Klugman KP Adrian PV Madhi SA Acquisition of Streptococcus pneumoniae in Pneumococcal Conjugate Vaccine-Naive South African Children and Their Mothers Pediatr Infect Dis J 2013 13 e192 e205 10.1097/INF.0b013e31828683a3 23340555

[B20] O'Brien KL Nohynek H Report from a WHO Working Group: standard method for detecting upper respiratory carriage of Streptococcus pneumoniae Pediatr Infect Dis J 2003 13 e1 e11 12586987 10.1097/01.inf.0000049347.42983.77

[B21] Park IH Pritchard DG Cartee R Brandao A Brandileone MC Nahm MH Discovery of a new capsular serotype (6C) within serogroup 6 of Streptococcus pneumoniae J Clin Microbiol 2007 13 1225 1233 10.1128/JCM.02199-06 17267625 PMC1865839

[B22] Margolis E Yates A Levin BR The ecology of nasal colonization of *Streptococcus pneumoniae*, Haemophilus influenzae and Staphylococcus aureus: the role of competition and interactions with host's immune response BMC Microbiol 2010 13 59 10.1186/1471-2180-10-59 20178591 PMC2844402

[B23] Pettigrew MM Gent JF Revai K Patel JA Chonmaitree T Microbial interactions during upper respiratory tract infections Emerg Infect Dis 2008 13 1584 1591 10.3201/eid1410.080119 18826823 PMC2609881

