# Supplementary material for: Interrelationship of Streptococcus pneumoniae, Haemophilus influenzae and Staphylococcus aureus colonization within and between pneumococcal-vaccine naïve mother-child dyads
Source: BMC Infect Dis. 2013 Oct 17;13:483. doi: 10.1186/1471-2334-13-483 (PMC4015913; doi:10.1186/1471-2334-13-483)
Supplement: Additional file 1: Table S1 — Numbers of children and mothers with single, dual, triple carriage or no carriage, further stratified by HIV-exposure or maternal HIV-infection status. [file 1471-2334-13-483-S1.docx]

**Additional file 1: Table S1:** Numbers of children and mothers with single, dual, triple carriage or no carriage, further stratified by HIV-exposure or maternal HIV-infection status.

| **Carriage** | **Group** | **Average children age at visit (months)** | | | | | | | | |
| --- | --- | --- | --- | --- | --- | --- | --- | --- | --- | --- |
|  |  | **1.6** | **2.5** | **3.5** | **4.5** | **7.4** | **9.5** | **12.5** | **16.2** | **24.2** |
| *S.pneumoniae* | Children | 6 | 21 | 39 | 66 | 83 | 97 | 68 | 48 | 49 |
|  | HUU (HEU) | 1 (5) | 0 (21) | 18 (21) | 28 (38) | 47 (36) | 48 (49) | 36 (32) | 30 (18) | 29 (20) |
|  | Mothers | 5 | 11 | 14 | 25 | 25 | 24 | 21 | 18 | 24 |
|  | HIV- (HIV+) | 0 (5) | 2 (9) | 3 (11) | 12 (13) | 13 (12) | 12 (12) | 12 (9) | 11 (7) | 17 (7) |
| *S.aureus* | Children | 46 | 47 | 45 | 32 | 16 | 14 | 9 | 13 | 3 |
|  | HUU (HEU) | 10 (36) | 22 (25) | 23 (22) | 18 (14) | 9 (7) | 7 (7) | 5 (4) | 11 (2) | 1 (2) |
|  | Mothers | 65 | 74 | 94 | 99 | 80 | 84 | 94 | 85 | 82 |
|  | HIV- (HIV+) | 17 (48) | 27 (47) | 49 (45) | 64 (35) | 41 (39) | 45 (39) | 46 (48) | 47 (38) | 47 (35) |
| *H.influenzae* | Children | 1 | 6 | 10 | 8 | 8 | 15 | 13 | 17 | 15 |
|  | HUU (HEU) | 0 (1) | 2 (4) | 4 (6) | 4 (4) | 2 (6) | 6 (9) | 5 (8) | 7 (10) | 11 (4) |
|  | Mothers | 0 | 1 | 0 | 5 | 1 | 3 | 5 | 11 | 4 |
|  | HIV- (HIV+) | 0 (0) | 0 (1) | 0 (0) | 1 (4) | 0 (1) | 0 (3) | 0 (5) | 3 (8) | 1 (3) |
| *S.pneumoniae*  and *S.aureus* | Children | 6 | 20 | 22 | 33 | 13 | 12 | 16 | 9 | 12 |
|  | HUU (HEU) | 1 (5) | 5 (15) | 8 (14) | 22 (11) | 9 (4) | 10 (2) | 9 (7) | 5 (4) | 6 (6) |
|  | Mothers | 5 | 15 | 12 | 18 | 18 | 26 | 19 | 23 | 18 |
|  | HIV- (HIV+) | 0 (5) | 3 (12) | 6 (6) | 9 (9) | 9 (9) | 16 (10) | 13 (6) | 11 (12) | 12 (6) |
| *S.pneumoniae*  and *H.influenzae* | Children | 3 | 8 | 16 | 29 | 53 | 49 | 66 | 82 | 73 |
|  | HUU (HEU) | 1 (2) | 3 (5) | 6 (10) | 10 (19) | 27 (26) | 28 (21) | 31 (35) | 38 (44) | 41 (32) |
|  | Mothers | 2 | 1 | 1 | 1 | 0 | 4 | 1 | 1 | 0 |
|  | HIV- (HIV+) | 0 (2) | 0 (1) | 1 (0) | 1 (0) | 0 (0) | 2 (2) | 0 (1) | 0 (1) | 0 (0) |
| *H.influenzae*  and *S.aureus* | Children | 1 | 3 | 8 | 5 | 0 | 2 | 3 | 2 | 0 |
|  | HUU (HEU) | 0 (1) | 2 (1) | 5 (3) | 3 (2) | 0 (0) | 1 (1) | 3 (0) | 0 (2) | 0 (0) |
|  | Mothers | 0 | 1 | 2 | 2 | 3 | 1 | 5 | 8 | 7 |
|  | HIV- (HIV+) | 0 (0) | 0 (1) | 0 (2) | 0 (2) | 1 (2) | 0 (1) | 0 (5) | 3 (5) | 3 (4) |
| *S.pneumoniae* and  *H.influenzae* and  *S.aureus* | Children | 1 | 3 | 9 | 14 | 9 | 9 | 12 | 23 | 15 |
|  | HUU (HEU) | 0 (1) | 1 (2) | 3 (6) | 4 (10) | 3 (6) | 5 (4) | 2 (10) | 13 (10) | 7 (8) |
|  | Mothers | 0 | 0 | 0 | 2 | 2 | 1 | 4 | 3 | 1 |
|  | HIV- (HIV+) | 0 (0) | 0 (0) | 0 (0) | 0 (2) | 0 (2) | 0 (1) | 0 (4) | 1 (2) | 0 (1) |
| Non colonized | Children | 72 | 59 | 60 | 54 | 47 | 31 | 33 | 15 | 23 |
|  | HUU (HEU) | 11 (61) | 16 (43) | 22 (38) | 33 (21) | 18 (29) | 14 (17) | 22 (11) | 6 (9) | 14 (9) |
|  | Mothers | 59 | 64 | 86 | 89 | 100 | 86 | 71 | 60 | 54 |
|  | HIV- (HIV+) | 7 (52) | 19 (45) | 30 (56) | 35 (54) | 51 (49) | 44 (42) | 42 (29) | 34 (26) | 29 (25) |
| **Total** | Children | 136 | 167 | 209 | 241 | 229 | 229 | 220 | 209 | 190 |
|  | HUU (HEU) | 24 (112) | 51 (116) | 89 (120) | 122 (119) | 115 (114) | 119 (110) | 113 (107) | 110 (99) | 109 (81) |
|  | Mothers | 136 | 167 | 209 | 241 | 229 | 229 | 220 | 209 | 190 |
|  | HIV- (HIV+) | 24 (112) | 51 (116) | 89 (120) | 122 (119) | 115 (114) | 119 (110) | 113 (107) | 110 (99) | 109 (81) |

HUU – HIV unexposed uninfected children

HEU – HIV exposed uninfected children

A total of 1830 samples from mother-child dyads were analysed. In children, we give the total colonized (top row) and then the bottom row those colonized in HUU and HEU groups, i.e., HUU (HEU). Similarly in mothers, the top row gives the total colonized and the bottom row, those colonized per HIV-infection status.
